# Sub-acute intestinal obstruction – a rare complication of *Plasmodium falciparum* malaria in an adult: a case report

**DOI:** 10.1186/s13256-018-1730-z

**Published:** 2018-07-03

**Authors:** Tim Divine Bonghaseh, Domin Sone M. Ekaney, Michael Budzi, Gerald Ekwen, Steve Kyota

**Affiliations:** 1 Baptist Hospital Mutengene, Mutengene, Cameroon; 2 Clinical Research Education Networking and Consultancy (CRENC) Research Group, Douala, Cameroon; 3 Health and Human Development (2HD) Research Group, Douala, Cameroon; 4 Health Education and Research Organisation (HERO), Buea, Cameroon; 5 Faculty of Health Sciences, University of Buea, Buea, Cameroon

**Keywords:** *Plasmodium falciparum* malaria, Intestinal obstruction, Gastrointestinal manifestation, Case report

## Abstract

**Background:**

Malaria remains a major public health problem in most tropical countries. It occasionally presents with both typical and atypical signs and symptoms. Gastrointestinal manifestations are common in malaria endemic areas but intestinal obstruction as a complication is extremely rare.

**Case presentation:**

We present the case of a 42-year-old black African man who presented with signs and symptoms of intestinal obstruction and was diagnosed as having *Plasmodium falciparum* malaria. He was successfully treated with both parenteral and orally administered antimalarial medication and the intestinal obstruction subsequently resolved.

**Conclusion:**

With intestinal obstruction being an important cause of morbidity and mortality, we report this case to highlight this rare complication of malaria and therefore increase physicians’ awareness and prompt diagnosis and management.

## Background

Malaria is an endemic disease in sub-Saharan Africa. In Cameroon, *Plasmodium falciparum* (*P. falciparum*) has been reported to be responsible for 90–95% of all cases of clinical malaria [1]. The clinical presentation of malaria is variable with only 50–70% of patients presenting with classic paroxysms of fever [2]. The main symptoms of malaria include fever, headache, chills, rigors, sweats, anorexia, abdominal pain, nausea, vomiting, diarrhea, pallor, and jaundice [2]. Gastrointestinal manifestations of uncomplicated malaria are nonspecific. Several specific complications including gastrointestinal bleeding, splenic rupture, acute abdomen, and sub-acute intestinal obstruction have been reported among children with complicated malaria [3, 4]. The occurrence of sub-acute bowel obstruction as a complication of severe malaria is an uncommon finding in adults. One case of malaria complicated by intestinal obstruction in an adult was reported by the Food and Drug Administration in 2003 [5]. Therefore, in any febrile patient presenting with symptoms and signs of intestinal obstruction in an endemic area or with a recent travel history to an endemic area, malaria should be considered a differential diagnosis. Our aim, therefore, was to highlight this rare presentation of malaria in order to increase awareness and hence prompt diagnosis and management.

## Case presentation

A 42-year-old Cameroonian (black African) man with no relevant past medical history presented to our hospital with a 1-week history of fever and a 4-day history of no bowel movements. The fever was intermittent and worse in the evenings, and associated with headache, joint pains, and anorexia. He self-medicated an undocumented orally administered antimalarial medication but symptoms persisted. Three days later, he developed generalized abdominal pain, colicky in character, with a severity of 5/10 on a visual analogue scale. This was associated with progressive abdominal distension and an altered bowel pattern initially characterized by an inability to pass stool and flatus over 4 days but he denied any vomiting. A worsening of his symptoms prompted his visit to our hospital.

On examination, his mucous membranes were dry, his conjunctivae were pink, and his vital signs were normal. His abdomen was mildly distended with mild diffuse tenderness. There was neither guarding nor rebound tenderness. Percussion note was tympanic and bowel sounds were hyperactive. His rectum was empty per digital rectal examination and his prostate was not enlarged. The rest of the physical examination was unremarkable.

A rapid diagnostic (Alere™ Malaria Ag P.f, Abbott Rapid Diagnostics, USA) test for malaria was positive. Also, his complete blood count values were within normal ranges: white blood cell count of 6200 cells/uL, hemoglobin of 12.5 g/dL, and platelet count of 222,000 cells/uL. His metabolic panel was within normal range: serum sodium ion (Na^+^) of 140 mmol/L, serum potassium ion (K^+^) of 4.0 mmol/L, serum chloride ion (Cl^−^) of 98 mmol/L, serum creatinine of 0.9 mg/dL, alanine aminotransferase was 41.2 U/L, aspartate aminotransferase was 33.0 U/L, total bilirubin was 0.81 mg/dL, and alkaline phosphatase was 55.9 U/L. His human immunodeficiency virus serology was non-reactive. His rapid antibody test for typhoid fever was also non-reactive. There was no significant air fluid level or increased bowel gas on a plain abdominal radiograph and an abdominal ultrasound revealed mild free intra-abdominal fluid in all four quadrants.

We concluded on a working diagnosis of functional bowel obstruction secondary to malaria. He received three doses of intravenously administered quinine base infusion (600 mg in 500 ml of dextrose 5% to flow for 4 hours every 8 hours for 24 hours) and intravenously administered crystalloids (0.9% normal saline at rate of 1 liter 8 hourly for 48 hours) and was placed on “nothing by mouth.” He passed a small quantity of feces 48 hours after initiation of antimalarial medication, with complete resolution of the abdominal pain, distension, and bowel movements on the fourth day of hospital stay. A stool examination was done with no organisms identified. We then concluded on a final diagnosis of sub-acute intestinal obstruction secondary to malaria. He was then discharged on an orally administered artemisinin-based antimalarial combination (artemether/lumefantrine 20/120 mg, 4 tablets 12 hourly for 3 days). His 2-week follow up was unremarkable.

## Discussion

The key clinical feature in this case as evident from our patient’s history is that the symptoms and signs of malaria are vast and can mimic other disease processes. The classic presentation of paroxysms of fever associated with chills, rigors, anorexia, and arthralgia can precede other nonspecific symptoms such as abdominal pain, distension, and obstipation, therefore posing a diagnostic dilemma. With increasing resistance to antimalarial medications, which is principally due to the development of immunity and indiscriminate use of these medications as seen in our case presentation, these atypical presentations are becoming more frequent especially in malaria endemic regions [2].

Atypical gastrointestinal complications of malaria include hepatic encephalopathy, fulminant hepatic failure, splenic rupture, acute pancreatitis, acalculous cholecystitis, duodenal perforation, gastroenteritis, and retroperitoneal lymphadenopathy [2]. Malaria causing sub-acute bowel obstruction has previously been reported in an 18-month-old girl in India [3] and a 48-year-old man in the USA [5]. Intestinal obstruction secondary to mechanical compression by an enlarged spleen in an adult with benign tertian malaria has also been reported [6]; however, the spleen was not enlarged in our case.

Bowel obstruction can be dynamic (mechanical) or adynamic (functional). Functional obstruction may be characterized by absent peristalsis (paralytic ileus) or peristalsis can be present but in a non-propulsive form [7] due to a bowel wall or splanchnic nerve dysfunction [8]. The commonest causes of functional bowel obstruction include generalized peritonitis, acute pancreatitis, electrolyte abnormalities, postoperative ileus, and intra-abdominal infections such as acute appendicitis and acute pelvic inflammatory disease [8].

The precise mechanism through which malaria results in bowel disorders in both adults and children remains blurred. It has been stipulated that *P. falciparum* infection results in changes in the erythrocyte membranes, activating the coagulation cascade [9]. This activation of the coagulation cascade coupled with a deficiency of a disintegrin and metalloproteinase with a thrombospondin type 1 motif, member 13 (a von Willebrand factor cleaving protease), which is also a consequence of *P. falciparum* infection, can result in microvascular obstruction and consequently result in bowel wall ischemia [10]. Alternatively, microvascular obstruction and ischemia could result from cytoadherence and rosetting of erythrocytes during an infection with *P. falciparum* [2]. Taniguchi *et al.* in 2015 infected susceptible species of mice with *Plasmodium berghei* ANKA and found that infected mice had less content in the cecum and lower areas of the intestines than uninfected control mice did after 9 days of infection [11]. Also, they observed detachment of intestinal epithelium and occlusion of blood vessels coupled with shortening and destruction of intestinal villi in infected mice [11]. Although this has not been proven to occur during a *P. falciparum* infection, their findings can suggest that an infection with *Plasmodium* species can result in functional bowel obstruction due to bowel wall dysfunction from bowel wall ischemia.

## Conclusion

The clinical course of severe *Plasmodium* malaria may mimic bowel obstruction; therefore, in febrile patients presenting with symptoms and signs of bowel obstruction, especially in malaria endemic areas, work-up for malaria should be included in the basic investigations.

## References

[1] Kwenti TE, TDB K, Njunda LA, Latz A, Tufon KA, Nkuo-Akenji T. Identification of the *Plasmodium* species in clinical samples from children residing in five epidemiological strata of malaria in Cameroon. Trop Med Health. 2017;45(1) Available from: http://tropmedhealth.biomedcentral.com/articles/10.1186/s41182-017-0058-5. Accessed 15 Oct 2017. 10.1186/s41182-017-0058-5 PMC5471890 28630585

[2] Zaki S, Shanbag P. Atypical manifestations of malaria. Res Rep Trop Med. 2011;2 10.2147/RRTM.S13431. PMC6415643 30881176

[3] Dass R, Barman H, Duwarah SG, Deka NM, Jain P, Choudhury V. Unusual presentations of malaria in children: an experience from a tertiary care Centre in north East India. Indian J Pediatr. 2010;77(6):655–60. 10.1007/s12098-010-0055-8 20358313

[4] Dewanda NK, Midya M. Perforated duodenal ulcer in a child: an unusual complication of malaria. Med J Dr Patil Univ. 2015;8(2):261.

[5] Will you have Intestinal obstruction with Malaria? - eHealthMe [Internet]. Available from: https://www.ehealthme.com/cs/malaria/intestinal-obstruction/. Accessed 9 Nov 2017.

[6] Dao L. A Case of malarial splenomegaly causing symptoms of intestinal obstruction. Bol Hosp. 1943;42(2):26–34.

[7] Bailey H, RJM L, Williams NS, CJK B, O’Connell PR. Bailey & Love’s short practice of surgery. London: Hodder Arnold; 2008. p. 1188–90.

[8] Kulaylat MN, Doerr RJ. Small bowel obstruction [Internet]: Zuckschwerdt; 2001. Available from: https://www.ncbi.nlm.nih.gov/books/NBK6873/. Accessed 17 Oct 2017

[9] Tessier-Marteau A, Sl C, Grand F, Asfar P, Zandecki M, Macchi L. DIC and peripheral gangrene in a severe *Plasmodium falciparum* malaria: the coagulation-inflammation cycle with *Plasmodium falciparum* as a model. Ann Biol Clin (Paris). 2009;67(5):569–72. 10.1684/abc.2009.0367 19789130

[10] Masse E, Hantson P. *Plasmodium falciparum* Malaria Complicated by Symmetrical Peripheral Gangrene, Bowel Ischemia, Repeated Candidemia, and Bacteraemia [Internet]. Case Reports in Medicine. 2014. Available from: https://www.hindawi.com/journals/crim/2014/696725/. Accessed 22 Oct 2017. 10.1155/2014/696725 PMC4000630 24812562

[11] Taniguchi T, Miyauchi E, Nakamura S, Hirai M, Suzue K, Imai T, et al. *Plasmodium berghei* ANKA causes intestinal malaria associated with dysbiosis. Sci Rep. 2015;5:srep15699. 10.1038/srep15699. PMC4621605 26503461

